# Acid‐sensing ion channel 1a is involved in ischaemia/reperfusion induced kidney injury by increasing renal epithelia cell apoptosis

**DOI:** 10.1111/jcmm.14238

**Published:** 2019-02-22

**Authors:** Nana Song, Zhihui Lu, Jian Zhang, Yiqin Shi, Yichun Ning, Jing Chen, Shi Jin, Bo Shen, Yi Fang, Jianzhou Zou, Jie Teng, Xiang-Ping Chu, Linlin Shen, Xiaoqiang Ding

**Affiliations:** ^1^ Department of Nephrology Zhongshan Hospital, Fudan University Shanghai China; ^2^ Shanghai Medical Center of Kidney Shanghai China; ^3^ Shanghai Institute of Kidney and Dialysis Shanghai China; ^4^ Shanghai Key Laboratory of Kidney and Blood Purification Shanghai China; ^5^ Hemodialysis quality control center of Shanghai Shanghai China; ^6^ Department of Biomedical Sciences, School of Medicine University of Missouri ‐Kansas City Missouri; ^7^ Department of Physiology and Pathophysiology, School of Basic Medical Sciences Fudan University Shanghai China

**Keywords:** acid‐sensing ion channels, apoptosis, calcium, ischaemia/reperfusion injury, kidney, mitochondrial transmembrane potential

## Abstract

Acidic microenvironment is commonly observed in ischaemic tissue. In the kidney, extracellular pH dropped from 7.4 to 6.5 within 10 minutes initiation of ischaemia. Acid‐sensing ion channels (ASICs) can be activated by pH drops from 7.4 to 7.0 or lower and permeates to Ca^2+^entrance. Thus, activation of ASIC1a can mediate the intracellular Ca^2+^ accumulation and play crucial roles in apoptosis of cells. However, the role of ASICs in renal ischaemic injury is unclear. The aim of the present study was to test the hypothesis that ischaemia increases renal epithelia cell apoptosis through ASIC1a‐mediated calcium entry. The results show that ASIC1a distributed in the proximal tubule with higher level in the renal tubule ischaemic injury both in vivo and in vitro. In vivo, Injection of ASIC1a inhibitor PcTx‐1 previous to ischaemia/reperfusion (I/R) operation attenuated renal ischaemic injury. In vitro, HK‐2 cells were pre‐treated with PcTx‐1 before hypoxia, the intracellular concentration of Ca^2+^, mitochondrial transmembrane potential (∆ψm) and apoptosis was measured. Blocking ASIC1a attenuated I/R induced Ca^2+^ overflow, loss of ∆ψm and apoptosis in HK‐2 cells. The results revealed that ASIC1a localized in the proximal tubular and contributed to I/R induced kidney injury. Consequently, targeting the ASIC1a may prove to be a novel strategy for AKI patients.

## 1 INTRODUCTION

Ischaemia‐reperfusion injury (IRI) is the main cause of acute kidney injury (AKI) during kidney transplantation, cardiac surgery, etc Renal IRI is characterized by acute tubular necrosis. However, the mechanism through which renal damage occurs is not fully understood. There is no established therapy to accelerate the recovery and attempts at preventing AKI are not universally effective. Despite the reversibility, at least partial loss of renal function in most patients that survive, the mortality of AKI remains high (over 50%).

Acidic microenvironment is commonly observed in ischaemic tissue. In the kidney, the pH value of tissue dropped from 7.4 to 6.5‐7.0 within 10 minutes initiation of ischaemia followed by a further decrease as ischaemia progressed.^1^, ^2^ In vitro, pH value of culture medium decreased from 7.4 to 6.4‐6.5 after 4 hours of hypoxic treatment .^3^ Acid‐sensing ion channels (ASICs) represent an H^+^‐gated Na^+^ channel family and are activated by extracellular protons.^4^ To date, six ASICs subunits have been described in mammals: ASIC1a, ASIC1b, ASIC2a, ASIC2b, ASIC3 and ASIC4. Among these subunits, ASIC1a and ASIC3 assembled channels are high proton sensitive, which can be activated by pH drops from 7.4 to 7.0 or lower.^5^ However, ASIC1a is the only one that is permeable to Ca^2+^ besides Na^+^.^6^ Because of Ca^2+^ permeability, ASIC1a can be triggered by extracellular acid to mediate the intracellular Ca^2+^ accumulation and play crucial roles in apoptosis of neuron.^7^ The inhibition of ASIC1a could attenuate the cell apoptosis and ameliorate ischaemia induced brain injury.^8^ However, whether activation of ASIC1a is involved in ischaemia induced renal injury remains unclear. It was reported that ASICs was expressed in human normal kidney tubular epithelia cells with alterative levels in clear cell renal cell carcinoma and Henoch‐Schönlein purpura nephritis.^9^, ^10^

The aim of the present study was to test hypothesis that extracellular acidosis caused by ischaemia increases renal epithelia cell apoptosis through ASIC1a‐mediated calcium entry. Our results revealed that ASIC1a but not ASIC3 mediated ischaemia‐induced apoptosis is likely to be a new mechanism involved in the pathogenesis of renal IRI.

## 2 MATERIALS AND METHODS

### 2.1 Animals

Male C57BL/6 mice (16 weeks, weighing 25‐30 g) were obtained commercially (Animal Center of Fudan University, Shanghai, China) and housed in acrylic cages with shredded corn cob bedding in an acclimatized room (12/12 hours light/dark cycle; 22 ± 3°C) and provided with water and mouse breeder chow ad libitum, according to standard protocols for animal care. The procedures used were approved by the Institutional Animal Care and Use Committee of Fudan University and adhered strictly to the National Institutes of Health Guide for the Care and Use of Laboratory Animals.

### 2.2 I/R model in mouse and reagents pretreatment

Fifty‐six mice were randomly divide into nine groups: Sham, vehicle+I/R, PcTx1 (0.2 nmol/kg)+I/R, PcTx1 (2 nmol/kg)+I/R, PcTx1 (4 nmol/kg)+I/R, PcTx1 (10 nmol/kg)+I/R, APETx2 (30 nmol/kg)+I/R, APETx2 (60 nmol/kg)+I/R, APETx2 (120 nmol/kg)+I/R Renal ischaemia was performed in 16‐week‐old male C57BL/6 J mouse as previously described.^3^ Briefly, mice were anaesthetized by intraperitoneal injection of 4% phenobarbitone (10 μL/g body weight). After performing a midline laparotomy, bilateral renal pedicles were clamped for 35 minutes using an atraumatic vascular clamp and then perfused, after which the incision was closed. The sham‐operated group underwent the same surgical procedure without clamping of the renal pedicle. Rectal temperature was maintained at 37℃. Post‐surgical analgesic was achieved by subcutaneous injection of buprenorphine (0.1 mg/kg, every 12 hours). After reperfusion for 24 hours, blood and kidney were harvested.

Venom PcTx1 extracted from South American Tarantula (peptide institute, Japan) and APETx2 (peptide institute, Japan) were dissolved and diluted in ddH_2_O. PcTx1 (0.2, 2, 4, 10 nmol/kg body weight) or APETx2 (30, 60, 120 nmol/kg body weight) or ddH_2_O were administered by tail vein, before I/R surgery. Injection of ddH_2_O served as vehicle control. The choice of dose of PcTx1 and APETx2 was based on the Ref. [11].

### 2.3 H/R in vitro and cell treatments

Human proximal tubular cell line (HK‐2 cells, ATCC) were cultured in DMEM/F12 medium containing 10% FBS, 1 g/L insulin, 0.55 g/L transferrin, 0.67 mg/L selenium, 2 mmol L^−1^ glutamine, 100 U/mL penicillin and 100 mg/mL streptomycin, in a humidified atmosphere with 5% CO_2_ at 37°C until confluence. For hypoxia/reoxygenation, cells were cultured in a hypoxic atmosphere containing 1% O_2_, 94% N_2_, 5% CO_2_ (Air Liquide) for 6 hours and then transferred to the normoxic conditions 21% O_2_ and maintained for 30 minutes or 1 hour.

PcTx1 and APETx2 were dissolved and diluted in ddH_2_O. Cells were pre‐treated with PcTx1 (5, 25, 100, 500 ng/mL) or APETx2 (0.1, 1, 10, 100 µmol L^−1^) previous to H/R

### 2.4 Western blotting

Cells and renal tissues were homogenized in ice‐cold lysis buffer containing protease and phosphatase inhibitors. After being centrifuged at 12,000 *g* for 15 minutes at 4°C, the supernatant was collected. Western blotting was performed as previously described^12^: samples (60 μg protein per Lane) were loaded and separated on a sodium dodecyl sulphate‐polyacrylamide gel and transferred to a PVDF membrane. The membrane was blocked with 5% nonfat milk and incubated with the primary antibodies against ASIC1a (1:500), ASIC2a (1:500), ASIC3 (1:500), GAPDH (1:10000) overnight at 4°C, then incubated with HRP‐conjugated secondary antibodies, and developed by chemiluminescent Horseradish Peroxidase Substrate. Results were normalized to GAPDH.

### 2.5 Real‐time quantitative PCR

Total RNAs were extracted with Trizol reagent (Thermo Fisher Scientific, Waltham, MA, USA). First‐strand cDNAs were then synthesized by reverse transcription using oligo (dT) and Superscript II (TOYOBO, Kita‐ku, Osaka, Japan) according to the manufacturer's protocol. Polymerase chain reaction (PCR) reactions were performed using SYBR‐green PCR master mixture (TOYOBO, Japan). The target gene and their primer sequences are shown in Table 1. Relative levels of mRNA expression were normalized to GAPDH expression for each gene.

**Table 1:** Primer sets used for real‐time PCR

Gene	Sense Primer(5’‐3’)	Antisense Primer(5’‐3’)
human ASIC1	ATGGAAAGTGCTACACGTTCAA	GTTCATCCTGACTATGGATCTGC
mouse ASIC1	AGGGCTTTTGGGTGACATCG	CAGCCGGTGCTTAATGACCT
human GAPDH	GGAGCGAGATCCCTCCAAAAT	GGCTGTTGTCATACTTCTCATGG
mouse GAPDH	AGGTCGGTGTGAACGGATTTG	GGGGTCGTTGATGGCAACA

### 2.6 Assessment of serum creatinine and urea

Blood samples were taken through cardiac puncture. Serum creatinine and urea were measured using Quantichrom Creatinine Assay Kit and Urea Assay Kit.

### 2.7 MTT assay

Briefly, cells (5, 000 cells per well) were plated onto the 96‐well culture plates. Following the PcTx1 (5, 25, 100, 500 ng/mL) or vehicle treatment, cell viability was tested by the routine 3‐[4, 5‐dimethylthiazol‐2‐yl]‐2, 5 diphenyltetrazolium bromide (MTT, Beyotime Biotechnology) assay. Optical density (OD) of MTT was measured by a microplate reader at 490 nm.

### 2.8 Histopathological Examinations and Immunohistochemical Staining

Kidney slices were fixed in 10% formalin, embedded in paraffin, cut into 5‐μm sections, and stained with Periodic Acid Schiff (PAS) Stain Kit, cleaved‐caspase3 immunohistochemical staining or TUNEL staining. For PAS attaining, histologic injury scores were evaluated under light microscopy by a pathologist blinded to the origin of preparations and determined using a scoring system, as described in the previous study. Injury was scored according to the percentage of damaged tubules as follows: no injury (0), mild: less than 25% (1), moderate: less than 50% (2), severe: less than 75% (3) and very severe: more than 75% (4).

Immunohistochemical staining was performed as described previously.^13^ After incubated with first antibody of cleaved‐caspase3 (1:100), the reaction was detected with avidin‐biotin‐HRP complex immuno detects kit and examined with light microscopy. Relative optical density [(positive staining‐background)/background] of positive stained cells were calculated in six representative sections from each animal in a blinded manner to the treatment by the software of Image Measure Version 1.0 (Fudan University, Shanghai, China).

Apoptosis was detected using a TUNEL apoptosis assay kit (KeyGen Biotech, China) as previously described.^14^ Percentage of TUNEL positive staining area was measured under magnification ×400.

Immunofluorescence double staining was performed as described previously.^15^ After incubated in 0.3% BSA, the slices was incubated in the mixed primary antibodies: Guinea pig anti‐ASIC1a (1:50 alomone lab, Israel)+Rabbit anti‐aquaporin 1 (AQP1, 1:100, Abcam, UAS), Guinea pig anti‐ASIC1a (1:50)+Rabbit anti‐ Tamm‐Horsfall protein (THP, 1:100, Abcam, UAS), Guinea pig anti‐ASIC1a (1:50)+Rabbit anti‐Synaptopodin (SYN, 1:100, Abcam, USA), Guinea pig anti‐ASIC2a (1:50 alomone lab, Israel)+Rabbit anti‐AQP1(1:100), Guinea pig anti‐ASIC2a (1:50)+Rabbit anti‐THP(1:100), Guinea pig anti‐ASIC2a (1:50)+Rabbit anti‐SYN (1:100), Guinea pig anti‐ASIC3 (1:50 alomone lab, Israel)+Rabbit anti‐AQP1(1:100), Guinea pig anti‐ASIC3 (1:50)+Rabbit anti‐THP(1:100), Guinea pig anti‐ASIC3 (1:50)+Rabbit anti‐ SYN (1:100). After washed in PBS, the immuno reaction was detected by Alexa Fluor 488‐conjugated donkey anti‐Guinea pig IgG (1:200, Thermo Fisher, USA) and Alexa Fluor 594‐conjugated donkey anti‐rabbit IgG (1:200, Thermo Fisher, USA) and examined under a confocal microscope.

### 2.9 Flow cytometry

AnnexinV‐FITC/PI staining kit was used to access apoptosis of HK‐2 cells according to the manufacturer's instructions. Flow cytometry was carried out using a FACSCalibur (Becton Dickinson, Heidelberg, Germany) and analysed using FlowJo 10.0 software.

### 2.10 Intracellular calcium measurement

The HK‐2 cells were treated as above. The stock solutions of Fluo‐4, AM were diluted with HBSS that contained (mmol L^−1^): 138 NaCl, 5.3146 KCl, 0.3 NaH_2_PO4, 0.4 KH_2_PO4, 4.2 NaHCO_3_, 5.6 D‐glucose and 10 HEPES. The HK‐2 cells were loaded with 5 µmol L^−1^ Fluo‐4, AM at 37℃ for 30 minutes. Then, the investigated cells were washed by HBSS, and visualized under a confocal laser scanning microscope or measured by flow cytometry. The calcium‐dependent fluorescence was excited at 494 nm laser line and the fluorescence signal was acquired at 516 nm.

### 2.11 Mitochondrial membrane potential (∆ψm)

To determine the changes in mitochondrial trans‐membrane potential of cells, Cells were stained with JC‐1 dye according to the manufacturer's protocol.^16^ After treatment as mentioned above, HK‐2 cells were cultured within 5 µg/mL JC‐1 working solution at 37°C for 20 minutes. After rinsed with PBS for three times, flow cytometry was used to detect the quantitative analysis of the red and green fluorescence signal. Red fluorescence represents JC‐1 aggregates in normal mitochondria, whereas green fluorescence represents JC‐1 monomers. Percentage of green fluorescence signal was used to assess the damage of mitochondrial.

### 2.12 Statistics

Statistical analysis was carried out with Statistical Package for the Social Sciences version 16.0. Data are presented as mean ± SE. For comparison between two groups, two‐tailed, unpaired t‐tests were used. For multiple comparisons, one‐way ANOVA was applied followed by post hoc Student and Newman‐Keuls test where appropriate. The values of the score were presented as a class variable, analysed by Kruskal‐Wallis non‐parametric test. All comparisons were two‐tailed and *P* < 0.05 was considered significant.

## 3 RESULTS

### 3.1 Distribution of ASIC1a, ASIC2a and ASIC3 in the kidney

**Figure 1:**
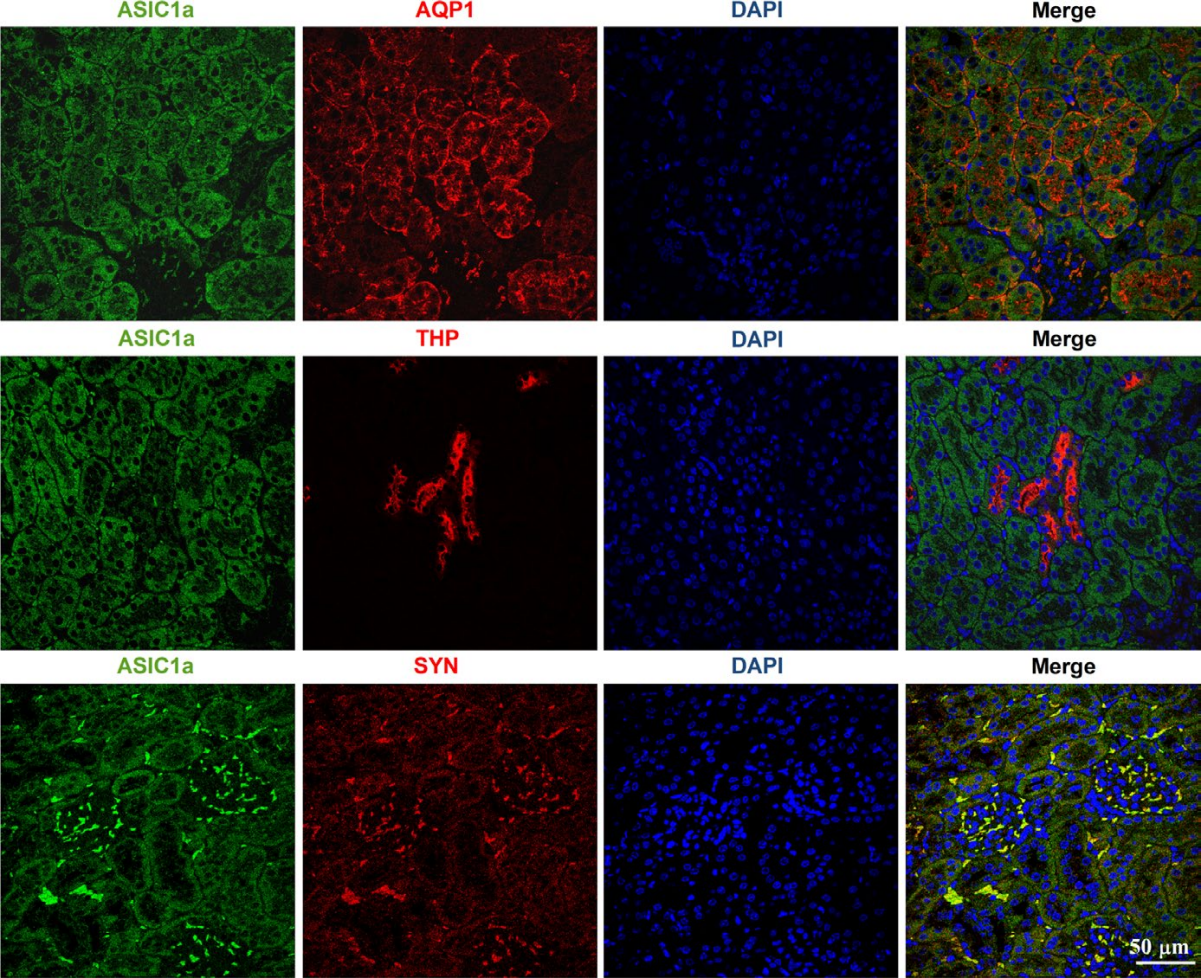
Immunohistochemical analysis of ASIC1a expression in renal tissue. Immunostaining for ASIC1a, AQP1, THP and SYN in renal tissue. ASIC1a was colocalized with AQP and SYN, but not THP. ASIC1a, acid sensing ion channel 1a; AQP1, aquaporin 1, proximal tubular cells marker; THP, Tamm‐Horsfall protein, thick ascending limb and distal tubular cells marker; SYN, Synaptopodin, podocyte marker; DAPI, 4,6‐diamidino‐2‐phenylindole, nuclear

To demonstrate the distribution of ASICs in the kidney, the colocalization between renal tubule and podocyte markers with ASIC1a, ASIC2a and ASIC3 in renal tissues was examined using immunohistochemistry and confocal microscopy. ASIC1a‐positive signals were observed in proximal cells (AQP1‐positive) and podocyte (SYN‐positive), but not in thick ascending limbs (THP‐positive) (Figure 1). ASIC2a‐positive signals were also widely distributed in kidney (Figure S1). Whereas ASIC3‐positive signals were present in podocyte and mesenchyma, few positive cells were detected in tubule (Figure S2).

### 3.2 Inhibiting ASIC1a by PcTx1 attenuate I/R induced kidney injury, in vivo

**Figure 2:**
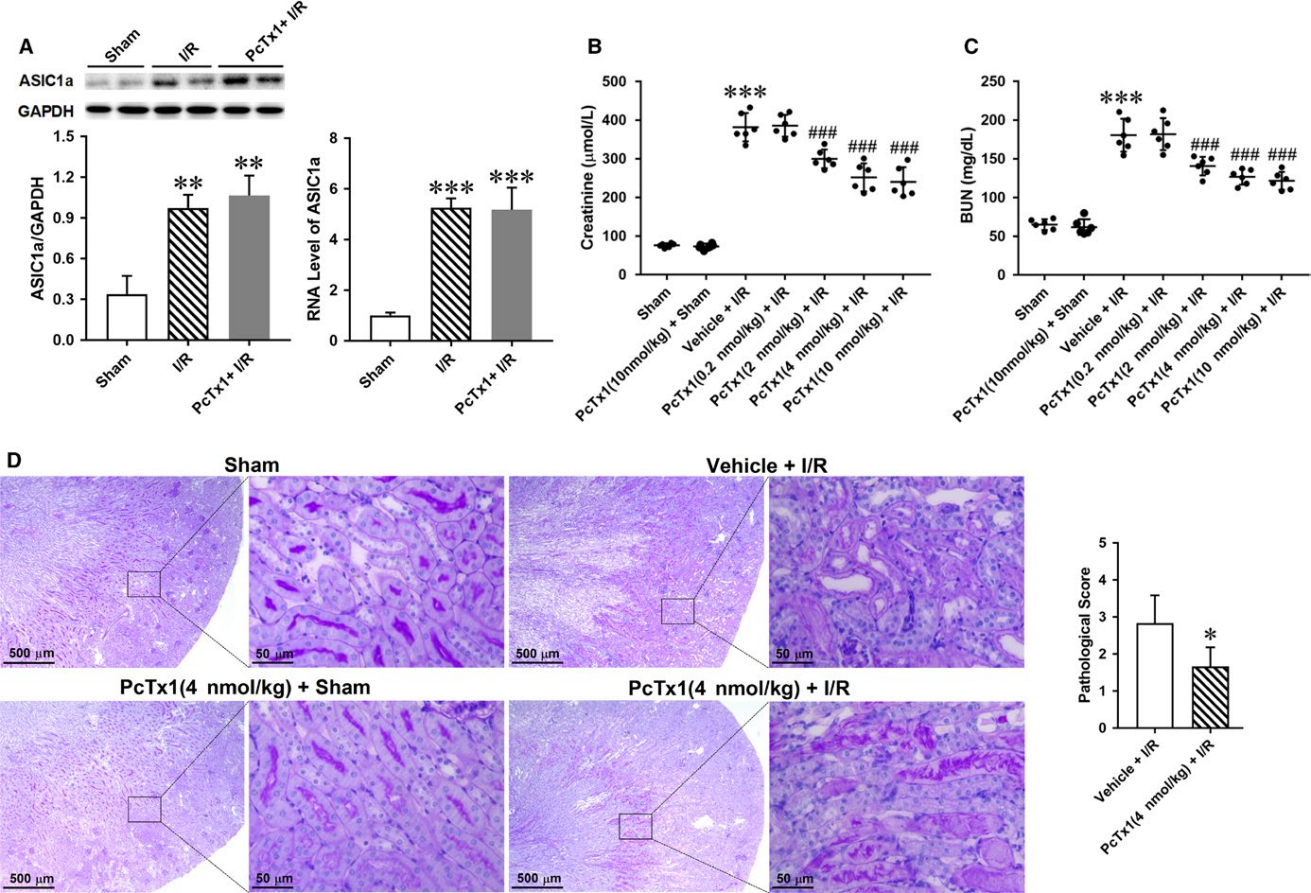
Inhibiting ASIC1a by PcTx1 attenuate I/R induced kidney injury. Different doses of PcTx1 (0.2, 2, 4, 10 nmol L^−1^ kg^−1^ body weight) or vehicle were injected by tail vein, 30 min before I/R operation. Twenty‐four hours after reperfusion, the expression of ASIC1a in the kidney of sham and I/R operating mouse was measured by Western blotting and PCR; functional and histological changes were assessed by serum creatinine, Urea and PAS staining; apoptosis of renal tubular epithelia cells was assessed by TUNEL staining. A, I/R increase protein and RNA level of ASIC1a in the kidney, however, administration of PcTx1 had no effect on expression of ASIC1a. B‐C, injection of PcTx1 reduced serum creatinine and urea in a dose dependent manner; D, Typical visual field of PAS staining and pathological score calculated from PAS staining. PcTx1 (4 nmol L^−1^ kg^−1^ body weight) significantly attenuated I/R induced renal injury. *P < 0.05, **P < 0.01, ***P < 0.001, compared with sham; ^#^ *P* < 0.05, ^###^ *P* < 0.001, compared with vehicle+I/R, n = 6

**Figure 3:**
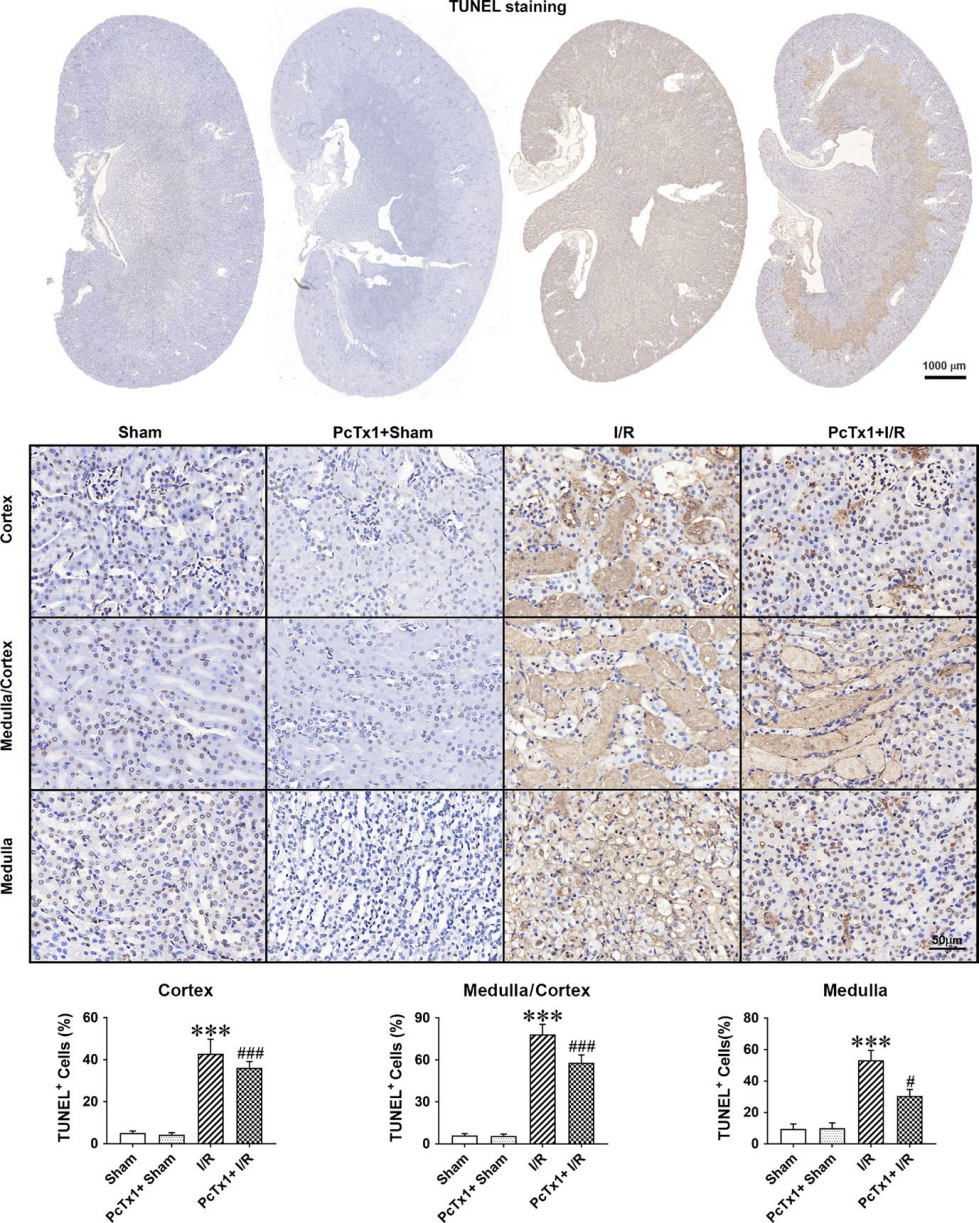
Inhibiting ASIC1a by PcTx1 attenuate I/R induced apoptosis of renal tubule, in vivo. Typical image of TUNEL staining in renal coronal section from sham, vehicle+I/R and PcTx1+ I/R animal. ***P < 0.001, compared with sham; ^#^ *P* < 0.05, ^###^ *P* < 0.001, compared with vehicle+I/R, n = 6

Renal ischaemic/reperfusion injury elevated the protein and RNA level of ASIC1a, while having no effect on that of ASIC2a or ASIC3 (Figure 2A and Figure S3A). To explore role of ASICs in I/R induced renal injury, different doses of ASIC1a inhibitor PcTx1 (0.2, 2, 4, 10 nmol/kg body weight) or ASIC3 inhibitor APETx2 (30, 60, 120 nmol/kg body weight) or vehicle was injected by tail vein before I/R operation. The renal function was assessed by serum creatinine and urea. Administration of PcTx1 reduced serum creatinine and urea in a dose dependent manner (Figure 2B‐C). However, injection of APETx2 had no effect on serum creatinine or BUN (Figure S3B). Additionally, PAS staining was performed to measure the renal injury. The results show that PcTx1 (4 nmol/kg body weight) significantly attenuated I/R induced renal injury, but APETx2 (120 nmol/kg body weight) did not (Figure 2D and Figure S3C‐D). Apoptosis of renal tubular epithelia cells was assessed by TUNEL staining. I/R operation caused apoptosis of renal epithelia cells and PcTx1 significantly attenuated I/R induced apoptosis (Figure 3). Importantly, injection of 4 nmol/kg PcTx1 in mice did not induce renal injury.

### 3.3 Inhibition of ASIC1a reduced H/R relative apoptosis of renal epithelia cells in vitro

**Figure 4:**
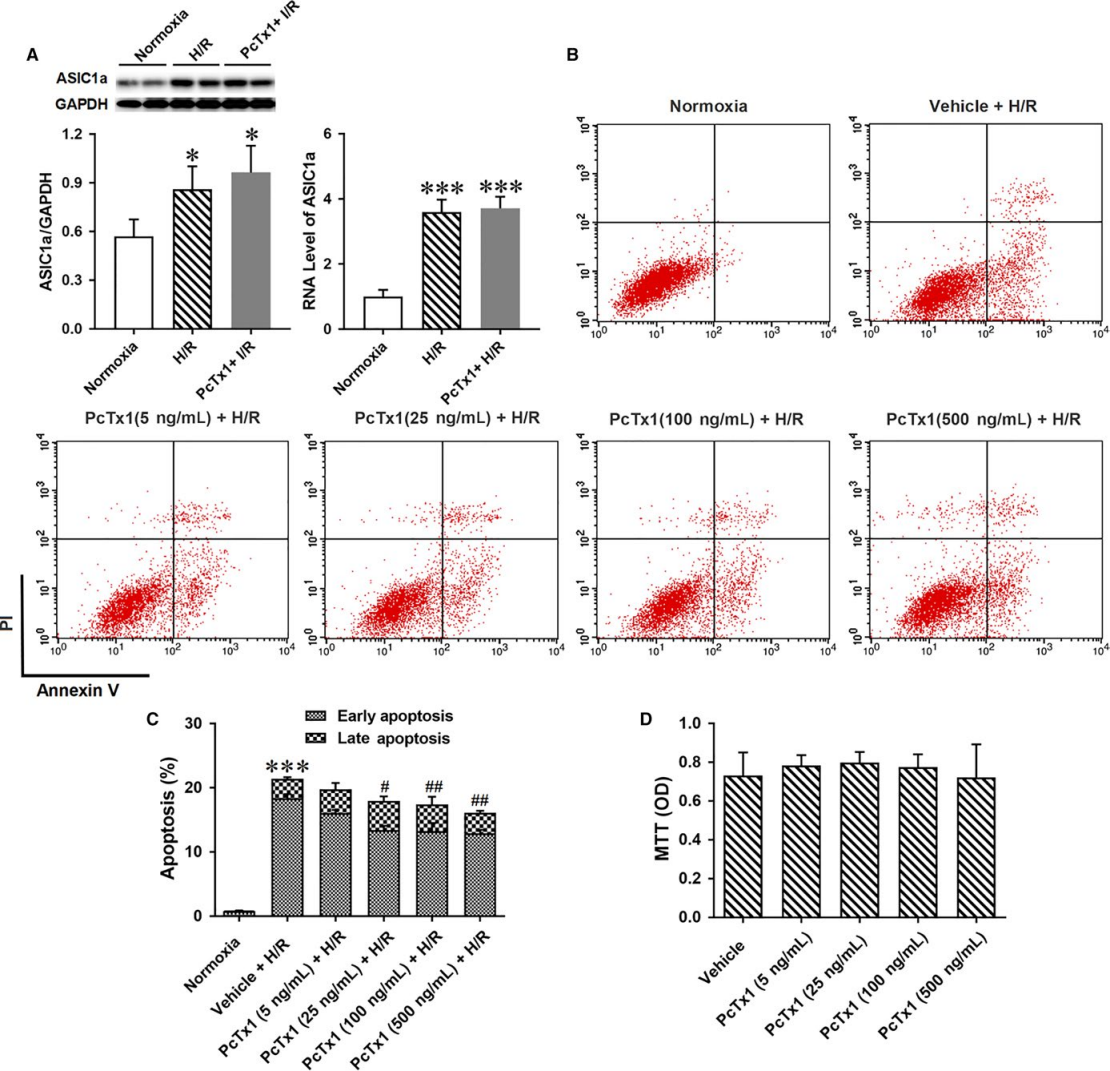
Inhibiting ASIC1a by PcTx1 reduced H/R induced apoptosis of HK‐2 cells. A, H/R increase protein level of ASIC1a in the HK‐2 cells. *P < 0.05, n = 6. B, HK‐2 cells were pre‐treated with different doses of PcTx1 (5, 25, 100, and 500 ng/mL) or vehicle before H/R treatment. Apoptosis of HK‐2 cells was measured by Annexin‐V/PI staining and evaluated by flow cytometry. C, the group data from B. PcTx1 attenuated H/R induced apoptosis especially early apoptosis dose dependently. ***P < 0.001, compared with Normoxia; ^#^ *P* < 0.05, ^##^ *P* < 0.01, compared with vehicle+H/R, n = 6. E, Cell variability was measured by MTT assay. Treatment of PcTx1 for 6 h had no effect on variability of HK‐2 cells

The expressions of ASICs in HK‐2 cells with or without hypoxia treatment were measured and compared. As expected, ASIC1a but not ASIC2a or ASIC3 in HK‐2 cells was upregulated by H/R (Figure 4A and Figure S4A).

Different doses of PcTx1 (5, 25, 100, 500 ng/ml) was applied to block ASIC1a, and APETx2 (0.1, 1, 10, 100 µmol L^−1^) was administered to block ASIC3 in HK‐2 cells before H/R treatment. Apoptosis of HK‐2 cells was measured by Annexin‐V/PI staining and evaluated by flow cytometry. PcTx1 attenuated H/R induced apoptosis especially early apoptosis does dependently. However, APETx2 had no effect on apoptosis (Figure 4B and C and Figure S4B). To determine whether cytotoxic effects of PcTx1 contributed to HK‐2 cell injury, cytotoxicity of PcTx1 was measured by MTT assay. Neither low nor high doses of PcTx1 affect cell variability (Figure 4D).

### 3.4 PcTx1 reduced H/R induced intracellular calcium overload and ∆ψm repression

**Figure 5:**
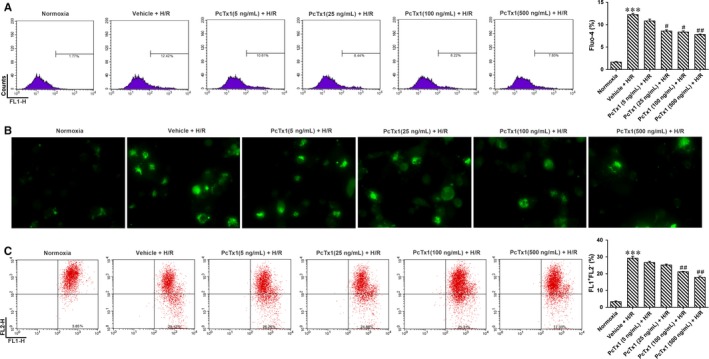
Inhibiting ASIC1a by PcTx1 reduced H/R induced intracellular calcium overload and loss of mitochondrial membrane potential. HK‐2 cells were administrated with different doses of PcTx1 as Figure 3. A, Intracellular calcium concentration were measured by fluo4 and evaluated by flow cytometry. PcTx1 attenuated H/R induced intracellular calcium overload dose dependently. B, Intracellular calcium concentration was measured by fluo4 immunofluorescence staining. As expected, PcTx1 attenuated H/R induced intracellular calcium overload. C, mitochondrial membrane potential were monitored by JC‐1 dye and evaluated by flow cytometry. ***P < 0.001, compared with Normoxia; ^#^ *P* < 0.05, ^##^ *P* < 0.01, compared with vehicle+H/R, n = 6

To explore whether the H/R induced apoptosis is mediated by the calcium permeability of ASIC1a, HK‐2 cells were treated with PcTx1 and intracellular calcium concentration were measured by fluo4, AM. H/R treatment increased intracellular calcium concentration ([Ca^2+^]_i_). However, PcTx1 attenuated [Ca^2+^]_i_ does dependently (Figure 5A and B).

Persistent increase in Ca^2+^ is a master cause of cell apoptosis. An excess rise in the [Ca^2+^]_i_ may result in decreased ∆ψm. Thus, ∆ψm was monitored by JC‐1 dye and evaluated by flow cytometry. Red fluorescence represents normal ∆ψm, whereas green fluorescence represents the loss of ∆ψm. The results indicated that H/R cause the loss of ∆ψm and PcTx1 protected mitochondria (Figure 5C).

### 3.5 Inhibiting ASIC1a reduced cleaved‐caspase3 in renal tubule

**Figure 6:**
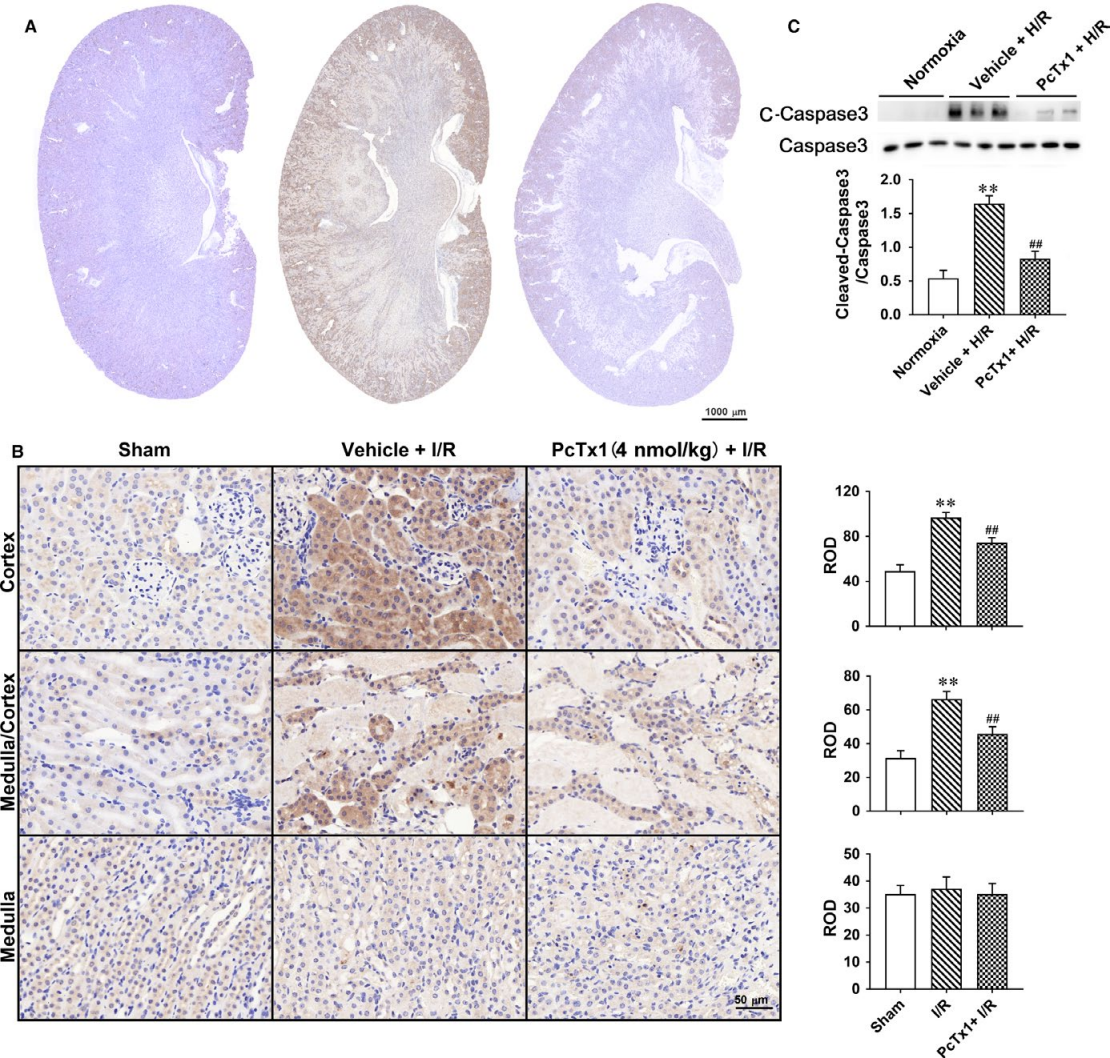
Inhibiting ASIC1a by PcTx1 reduced expression of cleaved‐caspase3 in renal tubule. A: PcTx1 (4 nmol L^−1^ kg^−1^ body weight) or vehicle were injected by tail vein, 30 min before I/R operation. Twenty‐four hours after reperfusion, apoptosis of renal tubular epithelia cells was assessed by immunohistochemical reaction of cleaved‐caspase3. Typical image of cleaved‐caspase3 immunohistochemical staining in renal coronal section from sham, vehicle + I/R and PcTx1+ I/R animal; B, detailed information of immunohistochemical reaction of cleaved‐caspase3 under high‐power lens; **P < 0.01, compared with sham; ^##^ *P* < 0.001, compared with vehicle + I/R, n = 6. C, HK‐2 cells were pre‐treated with PcTx1 (100 ng/mL) before H/R treatment, cleaved‐caspase3 was measured by Western blotting; **P < 0.01, compared with Normoxia; ^##^ *P* < 0.01, compared with vehicle+H/R, n = 6

cleaved‐caspase3 is the effector caspase required for morphological and biochemical process of apoptosis. The expression of cleaved‐caspase3 in the kidney was measured by immunohistochemical staining. The I/R operation elevated expression of cleaved‐caspase3, and blocking ASIC1a by PcTx1 attenuated activation of caspase3 (Figure 6A and B). The protein level of cleaved‐caspase3 in the HK‐2 cells was measured by Western blotting. The results show that H/R culture induced cleavage of caspase3. Pre‐treatment of PcTx1 suppressed the effect of H/R on caspase3 (Figure 6C).

## 4 DISCUSSION

Accumulating evidence demonstrated that ASICs participate in pathological progress of various organs such as brain, retinal, heart and articular cartilage ischaemic injuries.^17^, ^18^, ^19^, ^20^ ASIC1a a Ca^2+^‐permeable ASICs subunit serve as novel targets for ischaemic stroke therapy.^8^ PcTx‐1, a specific ASIC1a blocker is considered as a potential therapeutic strategy for stroke patients.^12^, ^21^ It was reported that ASIC1a was expressed in the human renal tubular epithelial cells and correlated with the development of Henoch‐Schönlein purpura nephritis.^10^ The kidney is extremely sensitive to ischaemic injury. However, it is unclear if ASIC1a participates in renal ischaemia injury. The overall aim of present study was to investigate how ASICs exerts its effects on renal IRI. We demonstrated that ASIC1a was expressed in proximal tubule cells and podocyte, ASIC2a was widely expressed in the kidney including tubule epithelia and podocyte, whereas ASIC3 was expressed in podocyte. In vitro and in vivo, ischaemia increased expression of ASIC1a but not ASIC3. Ischaemia‐mediated renal tubular injury is modulated by PcTx1 but not APETx2 a specific ASIC3 inhibitor. Moreover, we identified that ASIC1a mediated IR induced extracellular calcium entry and loss of mitochondrial membrane potential.

Acidosis is a detrimental condition accompanied with ischaemic injury. For example, in the brain, extracellular pH can fall to 6.5‐6.0 during ischaemia.^22^ And in the heart, ischaemia leads to severe myocardial acidification to pH 6.25 during 30 minutes of ischaemia.^23^ As for the kidney, pH reduced to 7.0‐6.5 after ischaemia.^1^, ^2^ In vitro, hypoxia decreased pH value of culture medium from 7.4 to 6.4‐6.5.^3^ It is well accepted that ischaemia leads to changes in extracellular pH and thus activation of ASIC channels. Among six subunits, ASIC1a and ASIC3 are most sensitive to extra cellular pH and can be activated when the pH falls below 6.9.^24^ The ischaemia induced acidosis of microenvironment is adequate to activate them. However, little is known whether ASICs in renal tubular epithelia cells can be activated during IR and the role of ASICs in renal IRI. In the present study, we observed the distribution of ASIC1a and ASIC3 in the kidney. We found that ASIC1a was mainly in proximal tubule cells and podocyte, and ASIC3 was in podocyte. It was well known that the proximal tubule is the primary target of ischaemic injury.^25^, ^26^ Moreover, renal IRI increased expression of ASIC1a in the kidney both in vivo and in vitro. However, no definite change of ASIC3 was found. Additionally, inhibition of ASIC1a by PcTx‐1 attenuated renal IRI, however, blocking ASIC3 by APETx‐2 had no effect on I/R induced injury. Our results indicated that ASIC1a may serve as a potential target to mediate the biology response to renal ischaemia.

Renal tubular epithelia cells apoptosis is a fundamental form of cell necrosis in renal IRI.^27^ Mechanisms of stimulating apoptosis across reperfusion have not been comprehensively identified. However, according to previous studies, activation of ASICs accounts for acid‐induced apoptosis.^28^ In brain, ischaemia‐induced acidosis causes neuronal injury dependent of the activation of ASICs.^29^ Blocking ASIC1a by PcTx1 blunted stroke induced neuron apoptosis.^21^ In adjuvant arthritis rats, blocking ASICs provides protection for the articular cartilage by inhibiting acid‐induced apoptotic injury.^30^, ^31^ In our research, we found that treatment with PcTx1 attenuated the I/R‐induced increase in the number of cells positive for cleaved‐caspase3 and TUNEL. The results indicate that ASIC1a is involved in the mechanism of I/R induced renal tubular epithelial apoptosis.

Activation of ASIC1a induces Ca^2+^ entry and leads to intracellular Ca^2+^ overload, which is a primary factor that causes cell apoptosis.^32^ It was reported that ASIC1a played an important role in the pathogenesis of diseases associated with ischaemic injury, including strokes, retinal ischaemia, etc.^4^, ^18^, ^33^, ^34^, ^35^ In the present study, we found that blocking ASIC1a is protectable to renal ischaemic injury. Ischaemic injury to renal tubular epithelia is associated with increased Ca^2+^ uptake.^36^ AISC1a is permeable to Ca^2+^. Additionally, activation of ASIC1a could stimulate downstream Ca^2+^ influx pathways including NMDA and AMPA/kainite receptors and voltage‐gated Ca^2+^ channels and contribute to acidosis‐induced elevations in [Ca^2+^]_i_.^37^ Our data reveal elevations in [Ca^2+^]_i_ in H/R cultured HK‐2 cell, and inhibition of ASIC1a attenuates the [Ca^2+^]_i_ response. High [Ca^2+^]_i_ is deleterious to mitochondrial because the mitochondria is the primary buffer for [Ca^2+^]_i_. Intracellular calcium overload leads opening of the mitochondrial permeability transition pore and loss of Δψm, which is an early event in the apoptotic process.^38^ In the current study, I/R induced Δψm and this effect was counteracted by administration of PcTx‐1.

AQP1 is expressed in the proximal tubule and the thin descending loop of Henle, playing important roles in tubule water permeability and the mechanisms of counter‐current exchange in the kidney.^39^ Moreover, resent research found that the expression level of renal cortical AQP1 was significantly reduced by renal I/R.^40^ AQP1 facilitated migration of epithelial cells in proximal tubule and deficiency of AQP1 aggravate I/R induced tubule damage.^41^ Our results showed that ASIC1a was colocalized with AQP1, which indicated that ASIC1a was distributed in the proximal tubule and the thin descending loop of Henle. And ASIC1a was found to be involved in renal IRI. However, it was unclear if ASIC1a and AQP1 interact with each other in the process of I/R induced renal injury. Tamm‐Horsfall protein (THP) also known as uromodulin, expressed exclusively in thick ascending loop of Henle and distal tubule.^42^ THP protects the kidney from IRI and its absence resulted in more severe inflammation and worse renal function.^43^, ^44^ However, our result indicated that ASIC1a was not colocalized with THP. The mechanisms responsible for the ASICs in renal IRI remain to be clarified in future studies.

## 5 CONCLUSIONS

**Figure 7:**
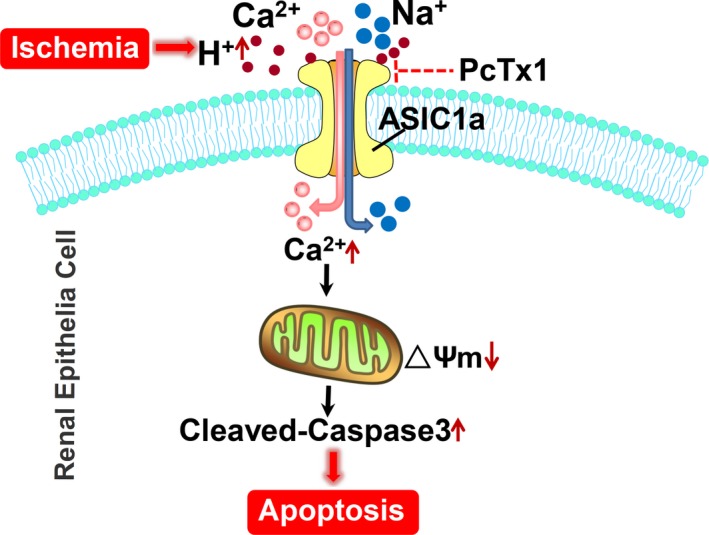
Schema of I/R induced the local microenvironment acidification and the activation of ASIC1a on renal injury and the involved underlying mechanism. Ischaemia caused accumulation of extracellular protons (H^+^). ASIC1a are activated by extracellular H^+^ and induce influx of Ca^2+^, which induced loss of mitochondrial membrane potential. The damage of mitochondrial increased cleaved‐caspase3, which resulted in apoptosis of renal tubular epithelia cells. Administration of the specific inhibitor of ASIC1a, PcTx1 ameliorated ischaemic renal injury by inhibiting ASIC1a, which implied a potential therapeutic choice for AKI

Acid‐sensing ion channels including ASIC1a, ASIC2a and ASIC3 are expressed in the kidney. ASIC1a mainly distributed in proximal tubule and ASIC3 are expressed in podocyte. ASIC1a appears to be involved in renal IRI. However, inhibition of ASIC3 had minor effect in renal IRI. Our studies demonstrate that ASIC1a inhibition is an important factor in protecting the kidney against IRI. Pharmacologic inhibition of ASIC1a could be a therapeutic strategy to protect the kidney in I/R This finding may provide strong evidence that ASIC1a are essential players in the pathophysiology of renal IRI. Also, the underlying mechanisms of ASIC1a in renal IRI are associated with its permeability to calcium (Figure 7). This mechanism and additional detailed mechanisms mediating renal IRI need further elucidation.

## CONFLICT OF INTEREST

The authors declare that there are no conflicts of interest associated with this study.
