# Supplementary figures and images for: Acid‐sensing ion channel 1a is involved in ischaemia/reperfusion induced kidney injury by increasing renal epithelia cell apoptosis
Source: J Cell Mol Med. 2019 Feb 22;23(5):3429–40. doi: 10.1111/jcmm.14238 (PMC6484315; doi:10.1111/jcmm.14238)

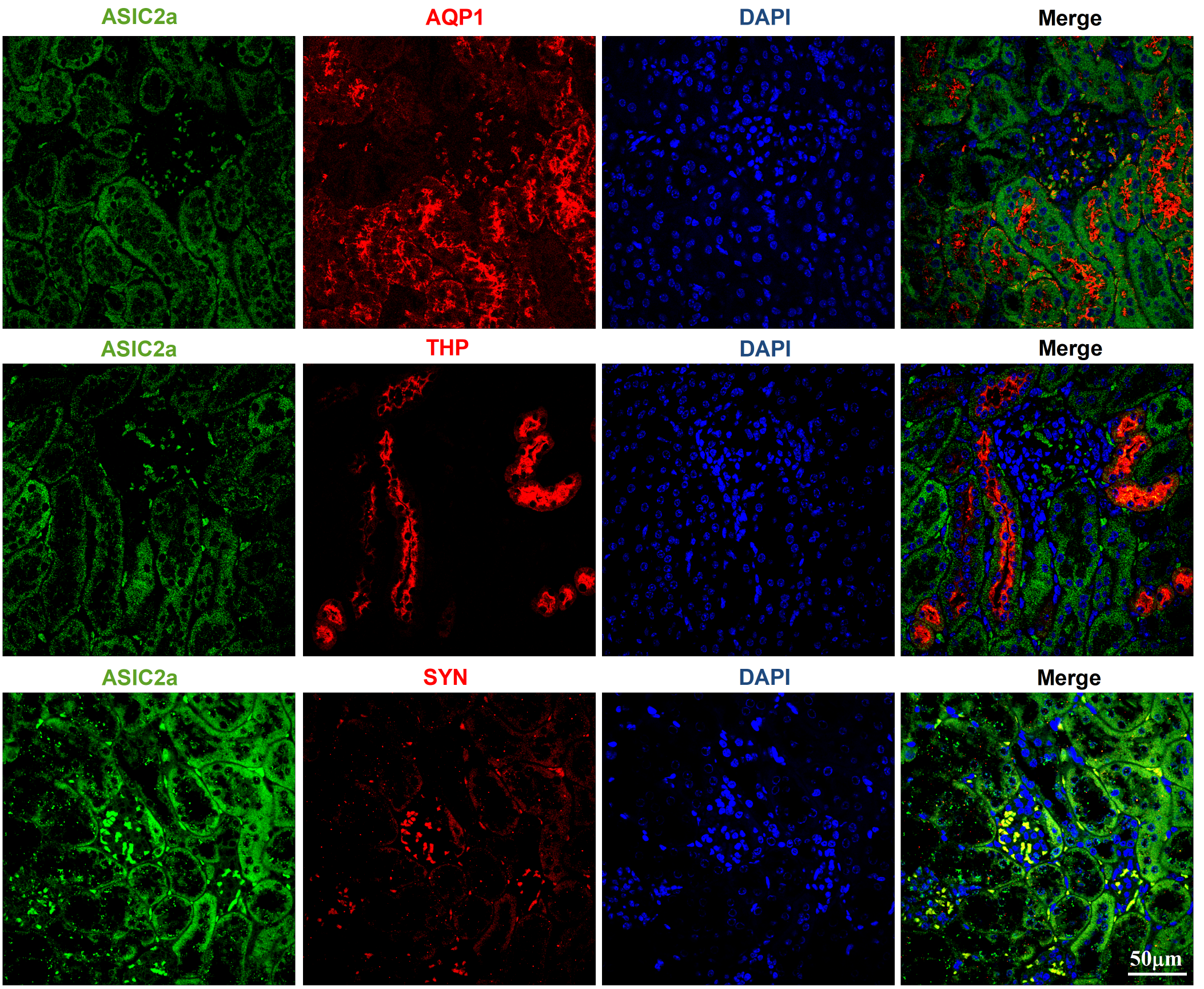

Supplement: Supplementary file 1 [file JCMM-23-3429-s001.tif]

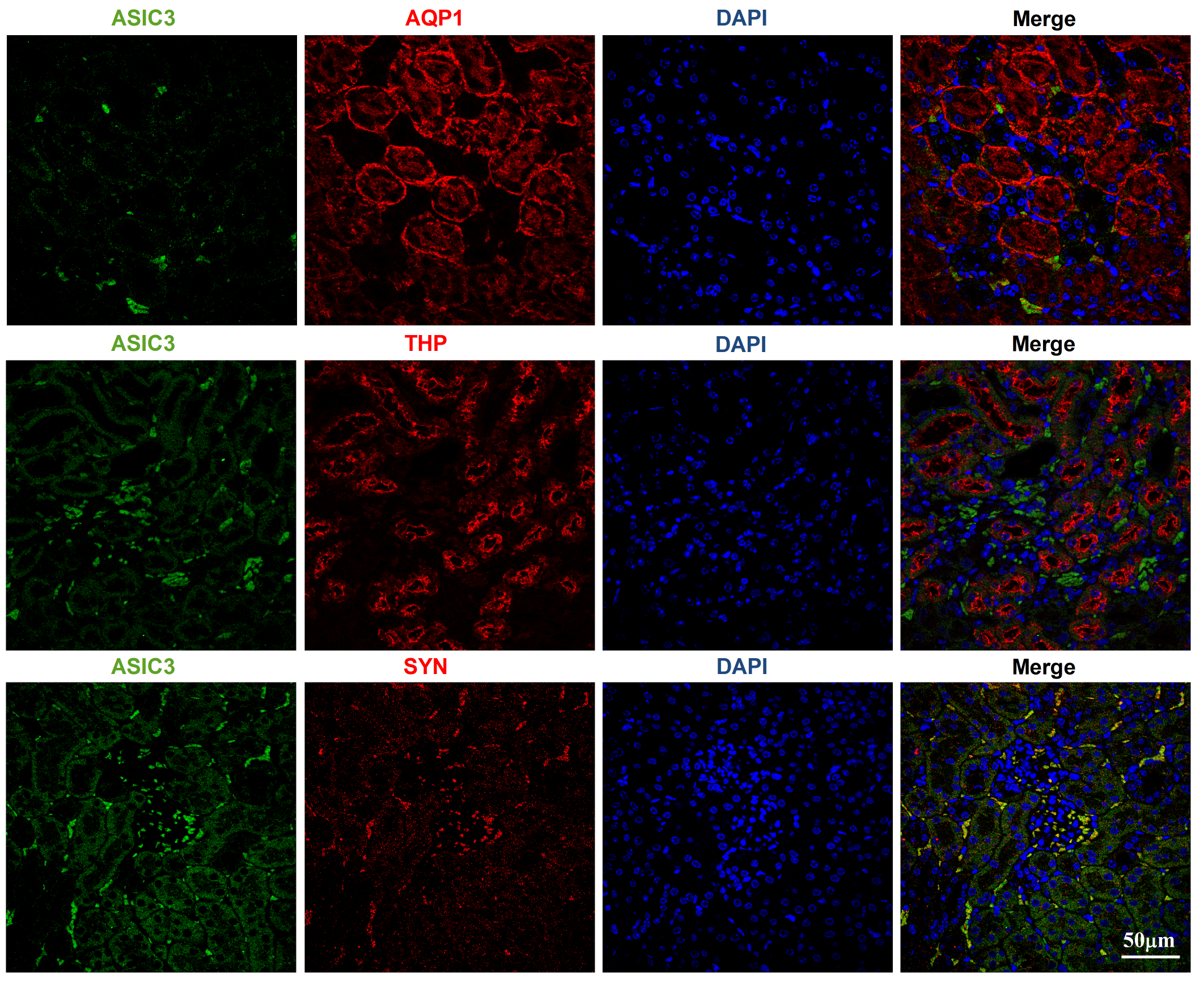

Supplement: Supplementary file 2 [file JCMM-23-3429-s002.tif]

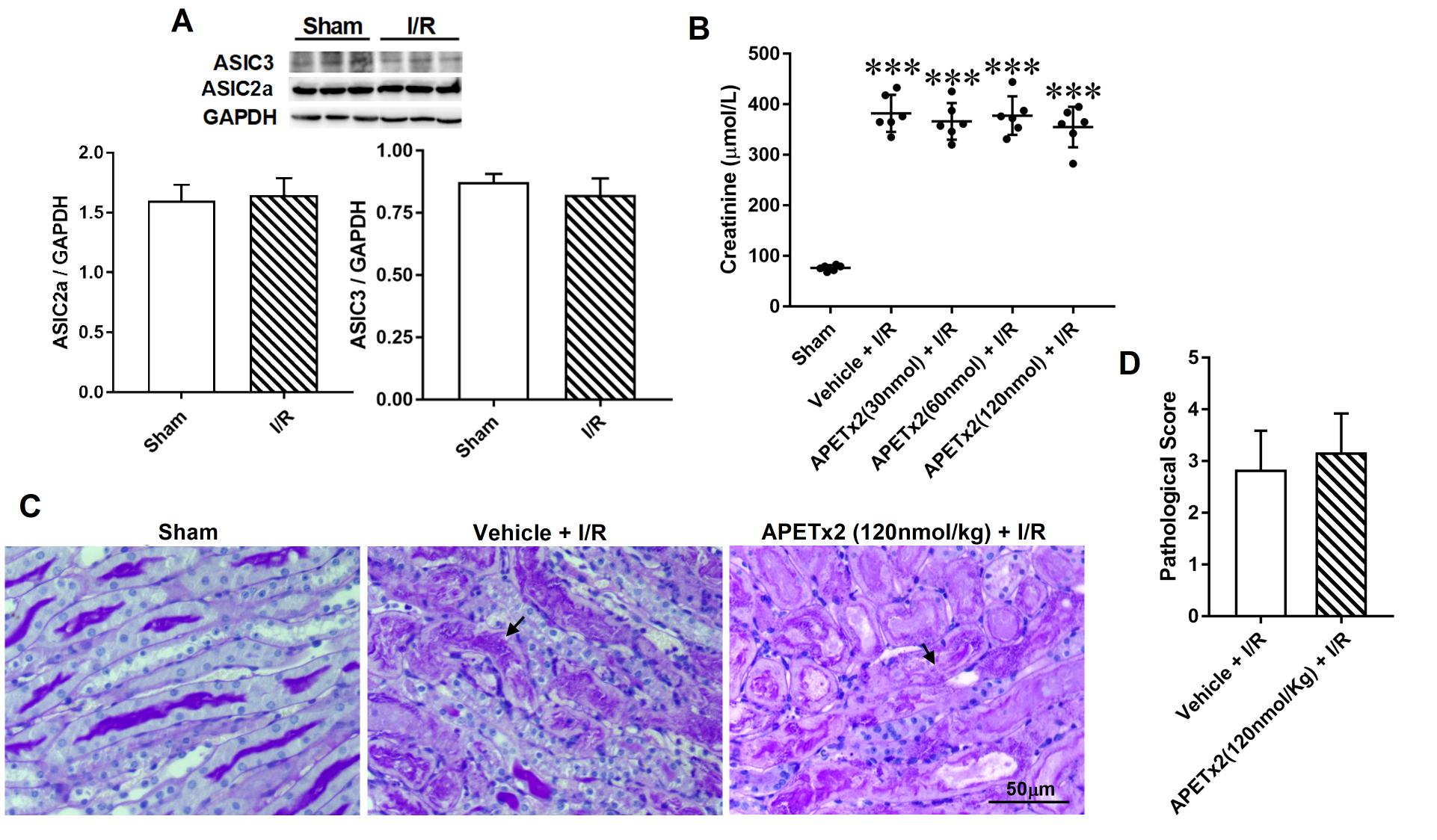

Supplement: Supplementary file 3 [file JCMM-23-3429-s003.tif]

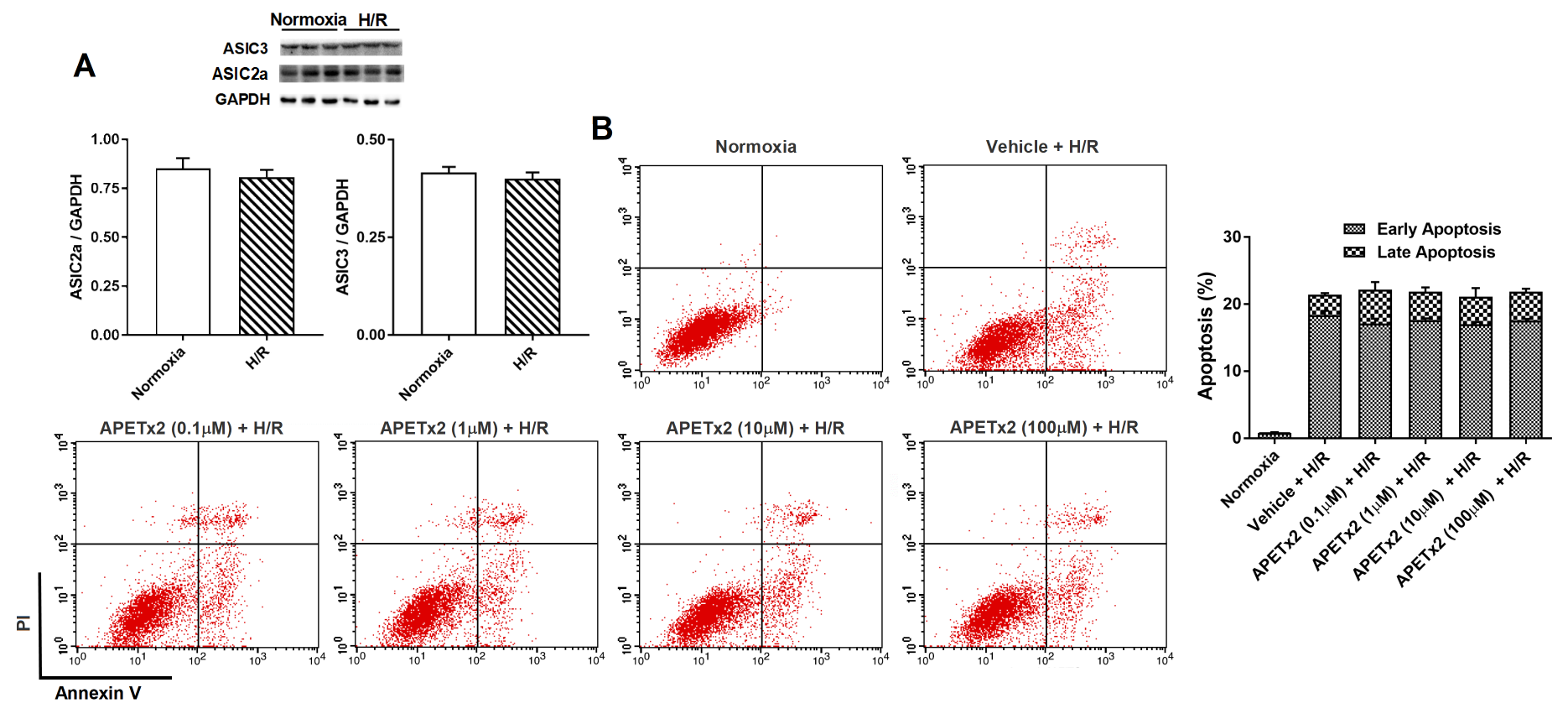

Supplement: Supplementary file 4 [file JCMM-23-3429-s004.tif]
